# Substance use‐related problems in mild intellectual disability: A Swedish nationwide population‐based cohort study with sibling comparison

**DOI:** 10.1002/jcv2.12225

**Published:** 2024-02-18

**Authors:** Andreas Påhlsson‐Notini, Shengxin Liu, Magnus Tideman, Antti Latvala, Eva Serlachius, Henrik Larsson, Tatja Hirvikoski, Mark J. Taylor, Ralf Kuja‐Halkola, Paul Lichtenstein, Agnieszka Butwicka

**Affiliations:** ^1^ Department of Medical Epidemiology and Biostatistics Karolinska Institutet Solna Sweden; ^2^ School of Health and Social Science Halmstad University Halmstad Sweden; ^3^ Institute of Criminology and Legal Policy University of Helsinki Helsinki Finland; ^4^ Department of Clinical Neuroscience Centre for Psychiatry Research Karolinska Institutet Stockholm Stockholm Sweden; ^5^ Department of Clinical Sciences Faculty of Medicine Lund University Lund Sweden; ^6^ School of Medical Sciences Örebro University Örebro Sweden; ^7^ Department of Women's and Children's Health Pediatric Neuropsychiatry Unit Center for Neurodevelopmental Disorders at Karolinska Institutet Karolinska Institutet Stockholm Sweden; ^8^ Habilitation and Health Stockholm Health Care Services Stockholm Sweden; ^9^ Center for Psychiatry Research Stockholm Sweden; ^10^ Division of Mental Health Services Akershus University Hospital and Institute of Clinical Medicine University of Oslo Oslo Norway; ^11^ Department of Biostatistics and Translational Medicine Medical University of Lodz Lodz Poland

**Keywords:** alcohol abuse, criminality, drug abuse, intellectual disability, substance use

## Abstract

**Background:**

Evidence for substance use‐related problems in individuals with mild intellectual disability is sparse and mainly limited to selected psychiatric populations. We evaluated the risk of substance use‐related problems in individuals with mild intellectual disability compared to the general population. Additionally, we have performed secondary sibling comparison analyses to account for familial confounding.

**Methods:**

We conducted a population‐based cohort study of individuals born in Sweden between 1973 and 2003. A total of 18,307 individuals with mild intellectual disability were compared to 915,350 reference individuals from the general population and 18,996 full siblings of individuals with mild intellectual disability. Information on mild intellectual disability and substance use‐related problems was obtained from several Swedish national and regional school and healthcare registers. Substance use‐related problems were measured via corresponding diagnostic and legal codes and included alcohol use disorder, drug use disorder, alcohol‐related somatic disease, conviction for a substance‐related crime, and substance‐related death.

**Results:**

Individuals with mild intellectual disability had a higher risk of any substance use‐related problem compared to the general population (HR, 1.81; 95% CI, 1.72–1.91), both in males (HR, 1.76; 95% CI, 1.65–1.89) and females (HR, 1.89; 95% CI, 1.74–2.05). The risks of substance use‐related problems were particularly elevated among individuals with mild intellectual disability and psychiatric comorbidities (HR, 2.21–8.24). The associations were attenuated in the sibling comparison models.

**Conclusions:**

Individuals with mild intellectual disability, especially those with psychiatric comorbidity, are at an elevated risk of substance use‐related problems. Familial factors shared by full siblings contribute considerably to the association between mild intellectual disability and substance use‐related problems.

**Key points**

- Individuals with mild intellectual disability are at increased risk of any of the included substance use‐related problems compared to the general population.
- Familial factors contribute considerably to the observed increased risk of substance use‐related problems.
- Both males and females with mild intellectual disability, especially those with psychiatric comorbidity, are at a similar and elevated risk of substance use‐related problems.
- Further development of prevention and treatment for substance use in individuals with mild intellectual disability is needed.

## INTRODUCTION

About 1% of the population worldwide is diagnosed with Intellectual disability (ID) and 85% of them have mild intellectual disability (MID) that is, a mild level of impairment (Maulik et al., 2011). ID is defined as a combination of impairment in general cognitive ability, impaired adaptive abilities, and early onset of symptoms (Boat & Wu, 2015). Substance use‐related problems (SUP) is a major contributor to the global public health burden, including substance use disorder, substance‐related crime, and substance use‐related death (Olusanya et al., 2020; Vos et al., 2020). SUP is prevalent in individuals with ID, posing challenges to the affected individual, society, healthcare and the criminal justice system (Carroll Chapman & Wu, 2012). A link between ID and increased risk of SUP has long been recognized in clinical settings, with a reported 30%–40% of individuals with substance use disorder also having comorbid ID or borderline intellectual functioning (N. van Duijvenbode & Van Der Nagel, 2019). The presence of substance use disorder can, however, camouflage the presence of ID in interactions with healthcare providers (Walker et al., 2022). The individuals with MID are disproportionally affected by SUP compared to the individuals with a more severe ID. Frequently discussed reasons for increased SUP in individuals with MID are higher cognitive and physical functioning, greater financial independence, greater participation in the community, and increased access to substances compared to individuals with more severe ID (Carroll Chapman & Wu, 2012). Current evidence on SUP in individuals with MID is predominantly limited to psychiatric or otherwise selected populations, and evidence from population‐based studies remains scarce, making general conclusions difficult (Didden, VanDerNagel, Delforterie, & van Duijvenbode, 2020). In addition, both MID and substance use disorder are more prevalent in males than in females, but potential sex differences in the association between them have been overlooked (McHugh et al., 2018; N. van Duijvenbode & VanDerNagel, 2019).

Moreover, individuals with MID are at increased risk of psychiatric comorbidity (Hirvikoski et al., 2021; Reppermund et al., 2019), further increasing the risk of substance use disorder and complicating effective treatment of substance misuse (Neomi van Duijvenbode et al., 2015). Yet, most existing studies on substance use disorder in ID have not illuminated the role of psychiatric comorbidity in the association.

Furthermore, evidence has suggested familial backgrounds in both ID and SUP (Khemiri et al., 2020; Latvala et al., 2016; Lichtenstein et al., 2021; Plomin & von Stumm, 2018). Sibling comparison design, by comparing exposed individuals to their unexposed full siblings from the same family, is widely used to account for unmeasured familial confounding, that is, genetics and shared environmental factors between siblings (Frisell et al., 2012). Thus, this nationwide cohort study aimed to evaluate the risk of SUP in individuals with MID compared to the general population. We also examined the role of sex and psychiatric comorbidities in this association. In addition, we performed sibling comparison analyses to account for the unmeasured familial confounding.

## METHOD

### Study design

This study was a population‐based cohort study with population and sibling comparisons using a matched cohort design using data from multiple Swedish registers (Table S1). By design, the sibling comparison design accounts for unmeasured factors, including both genetic and environmental elements that are shared among siblings. Specifically, full siblings share, on average, 50% of their genetic and stable environmental factors that persist across pregnancies and within the family context over time.

### Population

We identified individuals born in Sweden between 1973 and 2003 in the Medical Birth Register and followed them until 2013 using multiple Swedish nationwide and regional registers. Information on ID diagnosis was obtained from the National Patient Register (NPR), the Clinical Database for Child and Adolescent Psychiatry in Region Stockholm (PASTILL), the Habilitation Register (HAB), and the Halmstad University Register of Pupils with Intellectual Disability (HURPID), and based on the International Classification of Disease (ICD) codes or equivalents (Arvidsson, 2016; Brooke et al., 2017; Cnattingius et al., 1990; Idring et al., 2012; J. F. Ludvigsson et al., 2016; Jonas F Ludvigsson et al., 2011; Jonas F Ludvigsson et al., 2019; Långström et al., 2008; Wettermark et al., 2007).

### Inclusion and exclusion

**FIGURE 1:**
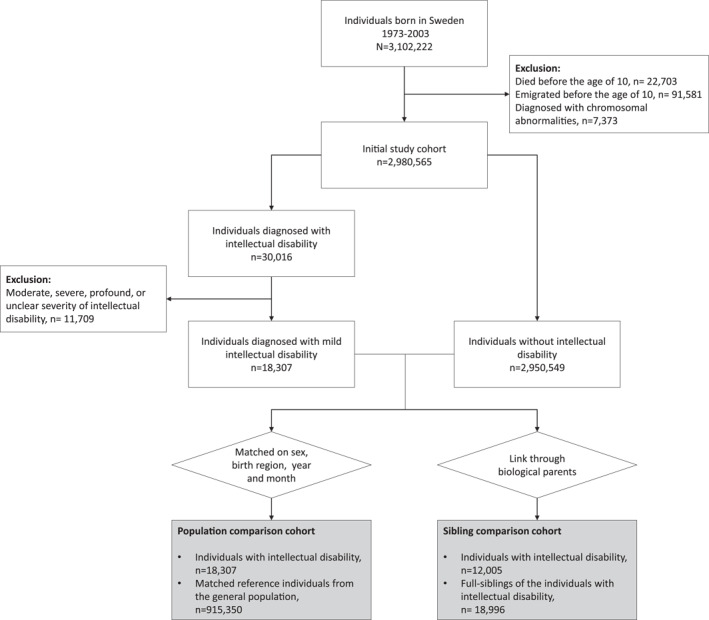
Study design.

We excluded individuals who died or emigrated from Sweden before the age of 10 years. We also excluded individuals diagnosed with chromosomal abnormalities (International Classification of Disease [ICD] codes in Table S2) due to their significant differences in terms of somatic multi‐morbidity, life expectancy, healthcare utilization and caregiver reliance (Jørgensen et al., 2019). We focused on the individuals with MID, that is, we did not include individuals with more severe (moderate, severe, or profound) or unclear severity of ID. This is because MID is identified as a risk factor for SUP, unlike the more severe forms of ID (Carroll Chapman & Wu, 2012; N. van Duijvenbode & VanDerNagel, 2019). This resulted in an initial study cohort of 2,980,565 individuals (Figure 1).

### Outcomes

We examined any SUP and specific SUP in terms of alcohol use disorder, drug use disorder, alcohol‐related somatic disease, conviction of a substance‐related crime, and substance‐related death which were gathered from NPR, National Crime Register and Cause of Death Register. Detailed specifications are provided in the Table S2.

### Comparisons

We estimated the risk of SUP for individuals with MID as compared to two reference groups: matched reference individuals from the general population (population comparison), and full siblings to individuals with MID (sibling comparison). The risks for any and specific SUP were also estimated in individuals with MID and a comorbid psychiatric disorder diagnosis compared to the same reference groups. For each individual with MID, we randomly selected 50 reference individuals without ID from the initial study cohort matched on sex, year and month of birth, and region of birth (population comparison). We also separately identified and matched their full siblings via the Multi‐Generation Register of individuals with MID (sibling comparison).

### Strata

Comorbid anxiety disorder, major depressive disorder, bipolar disorder, and psychotic disorder in individuals with MID were identified from NPR, while comorbid attention‐deficit/hyperactivity disorder (ADHD) and autism spectrum disorders (ASD) were identified from NPR, PASTILL, and HAB. Dispensed prescriptions of ADHD medication recorded in the Swedish Prescribed Drug Register were additionally used to identify individuals with ADHD. Diagnosis and Anatomical Therapeutic Chemical (ATC) classification codes are presented in Table S2.

### Covariates

The highest parental education level on record, a proxy of socioeconomic status, was acquired from the Longitudinal Integrated Database for Health Insurance and Labor Market Studies. Each consecutive 10 year period under study was designated as a separate birth cohort.

### Statistical analyses

We used Cox regression models with any and specific SUP separately to estimate the crude and adjusted hazard ratio (HR) with 95% confidence intervals (CI) for all outcomes between individuals with MID and the matched reference individuals from the general population (population comparison). The models were analyzed with each matched set entered as a stratum and adjusted for parental education attainment (population comparison). Missing parental education attainment was treated as a separate level. Crude and adjusted Cox regression models were also used to compare individuals with MID and their full siblings without ID (sibling comparison) with each family cluster entered as a stratum, and adjusted for sex and birth cohort. We repeated the analyses separately in males and females. We also repeated the analyses for individuals with comorbid MID and anxiety disorder, major depressive disorder, bipolar disorder, psychotic disorder, ADHD and ASD separately. Descriptive characteristics of the study populations are presented as numbers (%) for categorical variables. Hazard ratios for groups with *n* > 5 are presented as HR and 95% confidence interval. Statistical analyses were conducted using the survival package in *R*.

### Ethics approval

The study received approval from the Regional Ethical Review Board in Stockholm (2013/862‐31/5). We obtained anonymized data from Statistics Sweden and the National Board of Health Welfare. No informed consent was required.

## RESULT

A total of 18,307 individuals with MID (58.7% male), 915,350 reference individuals without ID, and 18,996 full siblings of individuals with MID were included in the study (Figure 1). Descriptive characteristics are presented in Table 1, age of first event for the SUP in Table S3 and information on the sibling cluster sizes in Table S4. Throughout the follow‐up, 8.1% of individuals with MID had any SUP event compared to 4.1% of the reference individuals.

**TABLE 1:** Baseline characteristics of individuals with mild intellectual disability (MID), matched reference individuals from the general population and full siblings of the individuals with MID.

	MID individuals	Reference individuals	Full siblings
N	18,307	915,350	18,996
Sex, n (%)			
Male	10,739 (58.7)	536,950 (58.7)	9649 (50.8)
Female	7568 (41.3)	378,400 (41.3)	9347 (49.2)
Birth cohort, n (%)			
1973–1983	3348 (18.3)	167,866 (18.3)	4654 (24.5)
1984–1993	9454 (51.6)	472,101 (51.6)	9210 (48.5)
1994–2003	5505 (30.1)	275,383 (30.1)	5132 (27.0)
Parental highest education level, n (%)			
Primary and lower upper secondary education	2429 (13.3)	43,913 (4.8)	2570 (13.5)
Upper secondary education	11,486 (62.7)	427,695 (46.7)	11,686 (61.5)
Post‐secondary education	4363 (23.8)	443,116 (48.4)	4724 (24.9)
Missing	29 (0.2)	626 (0.1)	16 (0.1)
Psychiatric comorbidities, n (%)			
Anxiety disorder	3375 (18.4)	58,020 (6.3)	1944 (10.2)
Major depressive disorder	1966 (10.7)	39,194 (4.3)	1218 (6.4)
Bipolar disorder	383 (2.1)	5479 (0.6)	174 (0.9)
Psychotic disorder	794 (4.3)	3969 (0.4)	162 (0.9)
ADHD	4972 (27.2)	32,279 (3.5)	1276 (6.7)
ASD	3437 (18.8)	12,065 (1.3)	505 (2.7)

Abbreviation: MID, Mild Intellectual Disability.

In the population comparison individuals with MID were at statistically significant increased risk of any SUP (HR, 1.82; 95% CI, 1.72–1.91) as evident in Table 2, and all examined specific SUP (HR ranging from 1.13 to 3.43), except for substance‐related death (Table S5). In the sibling comparison, the observed association was attenuated but remained statistically significant (HR, 1.37; 95% CI, 1.23–1.51) for any SUP, with specific SUP remaining significant for alcohol use disorder (HR, 1.31; 95% CI, 1.16–1.47) and drug use disorder (HR, 1.59; 95% CI, 1.37–1.86) as evident in Table S5.

**TABLE 2:** Incidence rates and risk estimates for any substance use‐related problem (SUP) by total and sex, in individuals with mild intellectual disability (MID) compared to matched reference individuals from the general population, and compared to full siblings.

	Population comparison				Sibling comparison			
	MID individuals, *n* (incidence rate^a^)	Reference individuals, *n* (incidence rate^a^)	Crude model, HR (95% CI)^b^	Adjusted model, HR (95% CI)^c^	MID individuals, *n* (%)	Full siblings, *n* (%)	Crude model, HR (95% CI)^d^	Adjusted model, HR (95% CI)^e^
All	1481 (3.5)	37,116 (1.7)	2.04 [1.94, 2.15]	1.82 [1.73, 1.91]	906 (3.2)	1121 (2.4)	1.41 [1.27, 1.56]	1.37 [1.23, 1.51]
Males	884 (3.6)	22,758 (1.8)	1.99 [1.86, 2.13]	1.77 [1.65, 1.89]	546 (3.4)	650 (2.4)	1.35 [1.18, 1.54]	1.26 [1.06, 1.49]
Females	597 (3.3)	14,358 (1.6)	2.12 [1.95, 2.30]	1.89 [1.74, 2.06]	360 (3.0)	471 (2.5)	1.49 [1.27, 1.74]	1.73 [1.40, 2.13]

Abbreviation: MID, Mild Intellectual Disability.

^a^ Incidence rate per 1000 person‐years.

^b^ Cox regression model with matched group as the stratum.

^c^ Additionally adjusted for parental educational attainment.

^d^ Cox regression model with family cluster as the stratum.

^e^ Additionally adjusted for sex and birth‐cohort.

The first event absolute incidence rate of any SUP per 1000 person‐years was 3.6 in males and 3.3 in females with MID, and both males (HR, 1.77; 95% CI, 1.65–1.89) and females (HR, 1.89; 95% CI, 1.74–2.06) were at increased risk of any SUP compared to reference individuals of the same sex (Table 2). An increased risk was observed for all SUP in both sexes, except for substance‐related death and alcohol‐related somatic disorder for females (Table S5). In the sibling comparison, the association between MID and any SUP was considerably attenuated among both males (HR, 1.26; 95% CI, 1.06–1.49) and females (HR, 1.73; 95% CI, 1.40–2.13).

The risk of any SUP in individuals with MID and a comorbid psychiatric disorder are presented in Table 3. Elevated risk of any SUP was observed for individuals with MID and each of the comorbid psychiatric disorders. The risk increase was greatest in individuals with MID and comorbid bipolar disorder (HR, 8.22; 95% CI, 6.81–9.92) and psychotic disorder (HR, 6.52; 95% CI 5.67–7.49), with a substantial increase in risk also in major depressive disorder (HR, 6.28; 95% CI, 5.74–6.87) and anxiety disorder (HR, 5.29; 95% CI, 4.92–5.70). The estimates for any and specific SUP were attenuated in the sibling comparison (Table S6).

**TABLE 3:** Incidence rates and risk estimates of any substance use‐related problem (SUP) by psychiatric disorder in individuals with mild intellectual disability (MID) compared to matched reference individuals from the general population, and compared to full siblings.

Psychiatric comorbidity	Population comparison^a^				Sibling comparison^b^			
	MID individuals, *n* (incidence rate^c^)	Reference individuals, *n* (incidence rate^c^)	Crude model, HR (95% CI)^d^	Adjusted model, HR (95% CI)^e^	MID individuals, *n* (incidence rate^c^)	Full siblings, *n* (incidence rate^c^)	Crude model, HR (95% CI)^f^	Adjusted model, HR (95% CI)^g^
Anxiety disorder	820 (9.6)	7587 (1.7)	5.98 [5.56, 6.43]	5.29 [4.92, 5.70]	502 (9.3)	318 (3.5)	2.97 [2.51, 3.51]	2.94 [2.48, 3.48]
Major depressive disorder	567 (11.2)	4484 (1.7)	7.10 [6.50, 7.76]	6.28 [5.74, 6.87]	352 (11.0)	212 (3.9)	3.46 [2.80, 4.26]	3.46 [2.79, 4.29]
Bipolar disorder	132 (13.2)	856 (1.6)	8.89 [7.38, 10.70]	8.22 [6.81, 9.92]	89 (13.7)	43 (3.8)	3.80 [2.47, 5.86]	3.80 [2.44, 5.91]
Psychotic disorder	233 (10.5)	1827 (1.5)	7.29 [6.35, 8.37]	6.52 [5.67, 7.49]	138 (10.1)	92 (3.9)	3.28 [2.37, 4.53]	3.30 [2.37, 4.58]
ADHD	668 (6.5)	8552 (1.6)	4.22 [3.90, 4.56]	3.77 [3.48, 4.09]	397 (6.2)	312 (3.0)	2.43 [2.03, 2.92]	2.44 [2.03, 2.94]
ASD	262 (3.6)	5702 (1.6)	2.36 [2.09, 2.67]	2.21 [1.95, 2.50]	168 (3.6)	178 (2.4)	1.76 [1.37, 2.27]	1.75 [1.35, 2.27]

Abbreviations: ADHD, Attention‐Deficit/Hyperactivity Disorder; ASD, Autism Spectrum Disorder; MID, Mild Intellectual Disability.

^a^ Reference individuals with or without psychiatric disorder.

^b^ Siblings with or without psychiatric disorder.

^c^ Incidence rate per 1000 person‐years.

^d^ Cox regression model with matched group as the stratum.

^e^ Additionally adjusted for parental educational attainment.

^f^ Cox regression model with family cluster as the stratum.

^g^ Additionally adjusted for sex and birth‐cohort.

The sex‐stratified model revealed gender‐specific risks of SUP in males and females with MID comorbid with neurodevelopmental disorders (Table 4). Females with MID and comorbid ADHD (HR, 4.56; 95% CI, 3.99–5.21) or ASD (HR, 3.29; 95% CI, 2.74–3.95) showed higher risks of any SUP than males with MID and comorbid ADHD (HR, 3.43; CI 95%, 3.11–3.80) or ASD (HR, 1.71; CI 95%, 1.44–2.02) compared to their respective same‐sex reference individuals. In contrast, the risks were similar for males with ID and comorbid anxiety disorder (HR, 5.19; CI 95%, 4.67–5.77) or major depressive disorder (HR, 6.38; 95% CI, 5.61–7.26) and females with comorbid anxiety disorder (HR, 5.39; CI 95%, 4.86–5.97) or major depressive disorder (HR, 6.20; 95% CI, 5.48–7.02).

**TABLE 4:** Incidence rates and risk estimates of any substance use‐related problem (SUP) by psychiatric disorder and sex in individuals with mild intellectual disability (MID) compared to matched reference individuals from the general population, and compared to full siblings.

Psychiatric comorbidity	Population comparison^a^				Sibling comparison^b^			
	MID individuals, *n* (incidence rate^c^)	Reference individuals, *n* (incidence rate^c^)	Crude model, HR (95% CI)^d^	Adjusted model, HR (95% CI)^e^	MID individuals, *n* (incidence rate^c^)	Full siblings, *n* (incidence rate^c^)	Crude model, HR (95% CI)^f^	Adjusted model, HR (95% CI)^g^
Males								
Anxiety disorder	402 (10.5)	3774 (1.9)	5.99 [5.40, 6.65]	5.19 [4.67, 5.77]	253 (10.2)	143 (3.4)	2.93 [2.30, 3.72]	2.52 [1.85, 3.42]
Major depressive disorder	273 (13.0)	2158 (1.9)	7.27 [6.41, 8.26]	6.38 [5.61, 7.26]	171 (12.5)	92 (3.9)	3.37 [2.48, 4.59]	2.67 [1.83, 3.90]
Bipolar disorder	58 (14.2)	391 (1.8)	8.56 [6.47, 11.32]	7.83 [5.90, 10.39]	42 (15.1)	19 (4.2)	2.55 [1.38, 4.71]	1.98 [0.88, 4.48]
Psychotic disorder	136 (11.6)	1100 (1.7)	7.31 [6.10, 8.75]	6.44 [5.36, 7.73]	78 (10.7)	49 (3.9)	2.97 [1.93, 4.56]	2.40 [1.42, 4.07]
ADHD	424 (6.3)	5929 (1.7)	3.86 [3.49, 4.26]	3.43 [3.11, 3.80]	253 (5.9)	206 (2.9)	2.15 [1.72, 2.69]	2.17 [1.61, 2.93]
ASD	138 (2.9)	3851 (1.6)	1.83 [1.54, 2.17]	1.71 [1.44, 2.02]	85 (2.8)	107 (2.2)	1.29 [0.91, 1.84]	1.25 [0.80, 1.95]
Females								
Anxiety disorder	418 (8.8)	3813 (1.5)	5.96 [5.39, 6.60]	5.39 [4.86, 5.97]	249 (8.5)	175 (3.6)	3.01 [2.38, 3.80]	3.53 [2.55, 4.87]
Major depressive disorder	294 (10.0)	2326 (1.5)	6.95 [6.15, 7.85]	6.20 [5.48, 7.02]	181 (9.8)	120 (3.9)	3.53 [2.64, 4.71]	4.06 [2.76, 5.97]
Bipolar disorder	74 (12.5)	465 (1.5)	9.17 [7.15, 11.75]	8.54 [6.64, 10.99]	47 (12.6)	24 (3.5)	5.36 [2.91, 9.89]	6.85 [2.86, 16.41]
Psychotic disorder	97 (9.3)	727 (1.3)	7.26 [5.87, 8.99]	6.67 [5.37, 8.29]	60 (9.4)	43 (3.9)	3.71 [2.27, 6.06]	4.34 [2.31, 8.16]
ADHD	244 (7.1)	2623 (1.5)	5.04 [4.41, 5.75]	4.56 [3.99, 5.21]	144 (6.8)	106 (3.2)	3.06 [2.24, 4.18]	3.78 [2.41, 5.95]
ASD	124 (5.0)	1851 (1.5)	3.50 [2.92, 4.20]	3.29 [2.74, 3.95]	83 (5.1)	71 (2.7)	2.45 [1.70, 3.55]	2.61 [1.63, 4.17]

Abbreviations: ADHD, Attention‐Deficit/Hyperactivity Disorder; ASD, Autism Spectrum Disorder; MID, Mild Intellectual Disability.

^a^ Reference individuals with or without psychiatric disorder.

^b^ Siblings with or without psychiatric disorder.

^c^ Incidence rate per 1000 person‐years.

^d^ Cox regression model with matched group as the stratum.

^e^ Additionally adjusted for parental educational attainment.

^f^ Cox regression model with family cluster as the stratum.

^g^ Additionally adjusted for sex and birth‐cohort.

## DISCUSSION

### Main findings

In this large‐scale population‐based register study we found that individuals with MID were at an increased risk of SUP, including alcohol use disorder, drug use disorder, alcohol‐related somatic disorder, and substance‐related crimes. The risk of SUP was even more elevated among individuals with MID and comorbid psychiatric disorders, especially bipolar disorder and psychotic disorder. The risk of any SUD in individuals with MID in the population comparison (HR, 1.82) and in the sibling comparison (HR, 1.37) corresponds to a 54% reduction in effect size. Risk estimates were noticeably attenuated in sibling comparisons, indicating that familial factors (genetic and/or shared environmental factors) play important roles in the observed associations.

We found a higher prevalence of SUP in the MID population under study (8.1%) than their matched reference individuals from the general population (4.1%), which agrees with results from previous survey and interview studies (N. van Duijvenbode & VanDerNagel, 2019). These findings are also in line with a Canadian population‐based study, where Lin et al. found a 6% prevalence of substance use disorder among adults with ID and a 4% prevalence in adults without ID (Lin et al., 2016). However, the study was limited by the identification of ID based on disability income benefits, which may include individuals with other disabilities. In contrast, a Swedish study by Axmon et al. observed that 2% of older adults with ID (aged 55 and over) received a diagnosis of a substance use disorder, which was significantly lower than the 4% in the general population (Axmon et al., 2018). This discrepancy may be attributed to the different sources of identifying individuals with ID and the use of the disability service register, which also consists of people with other disabilities, acquired brain injuries, and severe ID. In addition, ID alone is associated with adverse health problems and premature mortality, and the exclusion of individuals that died before age 55 may have led to survival bias (Hirvikoski et al., 2021). Neither of the studies mentioned above have examined substance‐related crime or death, which are important consequences of substance use disorder.

Familial confounding is especially important when studying the association of MID and SUP due to considerable familial loading (Kendler et al., 2019; Lichtenstein et al., 2021) and shared familial influences on both cognitive abilities and SUP (Khemiri et al., 2020; Latvala et al., 2016). Yet, previous studies were not able to consider confounding from familial factors. In the present study, we employed a sibling comparison design to account for familial confounding. The observed associations were attenuated in the sibling comparison analyses which suggests that familial confounding, that is, environmental and genetic factors shared between full siblings, considerably contributes to the elevated risk of SUP in individuals with MID.

In terms of sex differences, substance use disorder has historically been viewed as prevalent mainly in men, although the perceived gender gap is slowly narrowing (Brady & Randall, 1999; McHugh et al., 2018). Previous research has highlighted sex differences in the acute and long‐term consequences of substance use disorder and that females communicate a larger family, social, medical and psychiatric impairment than males (McHugh et al., 2018). The current study shows that the risk of SUP likely is similar across sexes while comorbid ADHD and ASD increase risk in females. All in all, this corroborates and augments previous studies in regard to sex differences.

A particularly high relative risk of SUP was observed among individuals with MID and a comorbid psychiatric disorder, especially for bipolar and psychotic disorder, but also major depressive and anxiety disorder as well as ADHD and ASD, suggesting that these subgroups of individuals with ID may be particularly vulnerable. The results are in accordance with findings showing a high prevalence of substance use among individuals with MID recruited from psychiatric settings (Chaplin et al., 2011; Didden, Embregts, van der Toorn, & Laarhoven, 2009). We also confirmed previous observation of an increased risk of SUP among MID individuals with ASD in comparison to the general population (Butwicka et al., 2017).

Our study has several strengths, including large sample size, nationwide coverage, and identification of individuals with ID informed by multiple sources covering healthcare, habilitation and education records. Sweden has a tax‐funded welfare system with universal free‐of‐charge access to education and healthcare for all residents (Wettergren et al., 2016). This setting minimizes selection bias which is important since previous reports have linked low socioeconomic status with both ID and SUP. In addition, the use of sibling comparison design accounted for the unmeasured familial confounding, which has not been addressed in existing literature.

The study also has limitations. The observational register‐based character of the study only allowed us to evaluate registered SUP. Since managing substance use disorder has been a shared responsibility between healthcare and social services in Sweden, there is a possibility that a set of individuals with SUP have no contact with the medical care system and subsequently cannot be captured using the examined registers. Also, individuals with MID may still experience barriers in access to healthcare, and as a consequence, their substance use disorder and MID may remain undiagnosed (Ali et al., 2013; Doyle et al., 2022; Whittle et al., 2018). Since levels of substance use disorder severity or levels of sanction after criminal conviction is not differentiated in analyses it may well be true that individuals with MID have less severe SUP than others, or vice versa. It is also likely that individuals with MID have an impaired ability to hide criminal activity from others making the convictions overrepresented in these individuals. These limitations may lead to both under and overestimating the association between MID and SUP.

Several implications for educational, social, and healthcare services can be derived from this study. First, individuals with MID would benefit from psychoeducation and early intervention programs to improve skills for coping with mental health problems, to prevent substance misuse and the development of substance use disorder. Second, prevention and treatment programs for substance use disorder should be accessible and adapted to the needs of individuals with MID. Third, we observed females with MID are at the same risk of SUP as males, and thus efforts should be made to include all sexes in prevention, screening, treatment and research programs. Fourth, substance use disorder treatment programs for MID individuals should consider the complex clinical presentation, including high psychiatric comorbidity and the increased risk for females with comorbid ADHD and ASD. Moreover, the important role of familial factors suggests that preventive and therapeutic interventions for SUP in individuals with ID should also consider the vulnerabilities and needs of family members.

Future research may consider further examining and disentangling the genetic, non‐shared and shared environmental components of SUD in individuals with MID using more sophisticated models, such as quantitative genetic studies and familial co‐aggregations studies with data on more distant relatives (half‐siblings and cousins). Establishing the causal direction of SUP and psychiatric disorder in general may also inform prevention interventions. It may also help analysis to include levels of severity and sanction as covariates to control for differences in severity of outcomes in individuals with and without MID. It would also be of value to conduct population‐based studies in other populations with registers available for research to enable generalization to global populations.

## CONCLUSIONS

Our findings showed that MID is associated with a higher risk of SUP for both sexes. Individuals with MID and comorbid psychiatric disorders may have an exceptionally high risk of SUP, highlighting the need for special attention to this vulnerable group. Evidence from sibling comparisons suggests that familial factors contributes to the observed association between MID and SUP. Future research, including molecular and quantitative genetic studies, is warranted to identify the role of genetic factors and shared environment in the association between MID and SUP.

## AUTHOR CONTRIBUTIONS

**Andreas Påhlsson‐Notini**: Conceptualization; Data curation; Formal analysis; Investigation; Methodology; Project administration; Software; Validation; Writing – original draft; Writing – review & editing. **Shengxin Liu**: Conceptualization; Methodology; Visualization; Writing – original draft; Writing – review & editing. **Magnus Tideman**: Conceptualization; Funding acquisition; Resources; Writing – review & editing. **Antti Latvala**: Conceptualization; Writing – review & editing. **Eva Serlachius**: Conceptualization; Resources; Writing – review & editing. **Henrik Larsson**: Conceptualization; Resources; Writing – review & editing. **Tatja Hirvikoski**: Conceptualization; Writing – review & editing. **Mark J. Taylor**: Conceptualization; Writing – review & editing. **Paul Lichtenstein**: Conceptualization; Funding acquisition; Resources; Writing – review & editing. **Agnieszka Butwicka**: Conceptualization; Funding acquisition; Methodology; Project administration; Supervision; Writing – original draft; Writing – review & editing.

## CONFLICT OF INTEREST STATEMENT

All authors have completed the Unified Competing Interest‐form and declare: Henrik Larsson reports receiving grants from Shire Pharmaceuticals; personal fees from and serving as a speaker for Medice, Shire/Takeda Pharmaceuticals and Evolan Pharma AB; and sponsorship for a conference on attention‐deficit/hyperactivity disorder from Shire/Takeda Pharmaceuticals and Evolan Pharma AB, all outside the submitted work. The other authors have no financial relationships relevant to this article to disclose.

## ETHICAL CONSIDERATIONS

The study received approval from the Regional Ethical Review Board in Stockholm (2013/862–31/5).

## Supporting information

Supporting Information S1

## Data Availability

Data sharing is not applicable to this article as no new data were created or analyzed in this study.
