# Supplementary material for: Substance use‐related problems in mild intellectual disability: A Swedish nationwide population‐based cohort study with sibling comparison
Source: JCPP Adv. 2024 Feb 18;4(2):e12225. doi: 10.1002/jcv2.12225 (PMC11143951; doi:10.1002/jcv2.12225)
Supplement: Supplementary file 1 — Supporting Information S1 [file JCV2-4-e12225-s001.docx]

**Supporting Information**

**Table S1.** Descriptions of Swedish registers used in the present study.

| **Register, coverage period** | **Summary of available information** |
| --- | --- |
| Cause of Death Register (CDR), 1952- | CDR contains information on the primary and contributory causes of death and date of death (Brooke et al., 2017). |
| Clinical Database for Child and Adolescent Psychiatry in Stockholm (PASTILL), 2001- | PASTILL provides data from child and adolescent psychiatric inpatient and outpatients care within Region Stockholm. ASD diagnosis in PASTILL has been shown to have a validity of 96% (Idring et al., 2012). |
| Habilitation Register (HAB), 1997- | HAB contains information on the usage of Region Stockholm Habilitation Services due to disability, including intellectual disability, autism spectrum disorder, and impairment in motor, vision or hearing (Idring et al., 2012). |
| Halmstad University Register on Pupils with Intellectual Disability (HURPID), 2001- | HURPID includes all pupils with intellectual disability admitted to special upper secondary schools including information on the date of graduation and the attended special education program: national programs (including special programs e.g. focusing on sports) and individual programs (adjusted for individual needs for pupils with severe ID, including vocational and activity training) (Arvidsson, 2016). |
| Longitudinal Integration Database for Health Insurance and Labor Studies (LISA), 1990- | LISA integrates annual data on the labor market, education sector and social sectors for all individuals living in Sweden (Jonas F Ludvigsson, Svedberg, Olén, Bruze, & Neovius, 2019). |
| Multi-Generation Register (MGR), 1932- | MGR links Swedish residents to their parents. This register enables identification of parents and siblings within family structures (Idring et al., 2012). |
| National Crime Register (NCR), 1973- | NCR is held by Swedish National Council for Crime Prevention and contains data on all convictions of crimes in Sweden, including details of the crime and the convictions date. It is estimated that 99.95% of the records within the crime register are linked to a person via personal identification number (Långström, Grann, Ruchkin, Sjöstedt, & Fazel, 2008). |
| National Patient Register (NPR), 1964- | NPR provides information on inpatient (nationwide coverage since 1987) and outpatients (since 2001) specialist health care facilities. Diagnoses, coded using the International Classification of Diseases (ICD 7-10). The validity of inpatient psychiatric diagnoses has been found to be up to 96% (Jonas F Ludvigsson et al., 2011). |
| Prescribed Drug Register (PDR), 2005- | PDR contains all prescribed drugs in Sweden according to the Anatomical Therapeutic Chemical (ATC) Classification (Wettermark et al., 2007). |
| Swedish Medical Birth Register (MBR), 1973- | MBR includes antenatal, delivery and pediatric records for 99% of all deliveries in Sweden. The register is periodically reviewed for quality (Cnattingius, Ericson, Gunnarskog, & Källén, 1990). |
| Total Population Register (TPR), 1961- | TPR contains information on life events, including birth, death, marital status and migration for Swedish population (J. F. Ludvigsson et al., 2016). |

**Table S2.** ICD-codes, ATC-codes and conviction codes used in the present study.

| **Exposure** | | | |
| --- | --- | --- | --- |
|  | **ICD-8 (1969-1986)** | **ICD-9 (1987-1996)** | **ICD-10 (1997-)** |
| **Mild intellectual disability** | 311 | 317 | F70 |
| **Covariates** | | | |
|  | **ICD-8 (1969-1986)** | **ICD-9 (1987-1996)** | **ICD-10 (1997-)** |
| **Anxiety disorder** | 300.0-300.3, 300.5-300.9, 307 | 300.0-300.3, 300.5-300.9, 306, 308, 309 | F40-F48 |
| **Major depressive disorder** | 296.0, 300.4 | 296B, 300E, 311X | F32,F34 |
| **Bipolar disorder** | 296.1, 296.2, 296.3 | 296A, 296C, 296D, 296E | F30,F31 |
| **Psychotic disorder** | 295, 297,298 | 295, 297, 298 | F20-F26, F28, F29 |
| **Autism spectrum disorder** | - | 299A | F84.0, F84.1, F84.5, F84.8, F84.9 |
| **Attention-deficit hyperactivity disorder** | - | 314 | F90 |
|  | **ATC-codes**: N06BA01, N06BA02, N06BA04, N06BA09 | | |
| **Outcomes** | | | |
|  | **ICD-8 (1969-1986)** | **ICD-9 (1987-1996)** | **ICD-10 (1997-)** |
| **Any substance use-related problem** | Any code from the following specific categories. | Any code from the following specific categories. | Any code from the following specific categories. |
| **Alcohol use disorder** | 291, 303 | 291, 303, 305A | F10 |
| **Drug use disorder** | 304 | 304 | F11-F16, F19 |
| **Alcohol-related somatic disorder** |  |  |  |
| Disorders explicitly linked to alcohol misuse | 57100,57101 | 357F, 425F, 535D, 571A-571D | G31.2, G62.1, I42.6, K29.2, K70, K85.2, K86.0 |
| Liver and pancreatic diseases with alcohol misuse | 571 or 577 with 291 or 303 | 571 or 577 with 291, 303 or 305A | K29 with F10, K71-K74 with F10 |
| Gastritis with alcohol misuse | 535 with 291 or 303 | 535 with 291, 303 or 305A | K70 with F10, K85.2 with F10, K86 with F10 |
| Polyneuropathy with alcohol misuse | - | 357 (excluding 357A, 357W) with 291, 303 or 305A | G62 with F10 |
| Cardiomyopathy with alcohol misuse | 425 with 291 or 303 | 425 with 291, 303 or 305A | I42 with F10 |
|  |  |  |  |
| **Alcohol- or drug-related death** |  |  |  |
| Death due to alcohol use disorder | 303 | 303, 3050 | F10 |
| Death due to alcohol poisoning | E860, N980.0 | E8600, E8601 | X45, X65, Y15 with T51.0 |
| Death due to alcohol psychosis | 291 | 291 | F10.5 |
| Death due to liver disease and pancreatitis | 5710 | 5710-5713 | K70, K85.2, K86 |
| Death due to drug dependence and poisoning | 304.0-304.1, 304.3-304.9, E853.0, E856.4, E859.0 | 292, 304.0, 304.2-9, 305.2-3, 305.5-7, 305.9, E850.0, E854.1-2, E855.2 | F11-F16, F19, T40.0, T40.2-T40.3, T40.5-T40.7, T40.9, T43.6 |
| **Substance use-related criminality** | **Law sections for criminal convictions** | | |
| Alcohol | Alkohollag (1994:1739) 2, 3, 4, 10 kap.  Alkohollag (2010:1622) 3, 11 kap. | | |
| Drugs | Narkotikastrafflag (1968:64)  Lag om kontroll av narkotika (1992:860) | | |
| Driving under the influence | Lag om straff för vissa trafikbrott (1951:649) 4, 4a § | | |
| Smuggling | Lag om straff för smuggling (2000:1225) 6 § | | |
| Doping | Lag om förbud mot vissa dopningsmedel (1991:1969) | | |
| **Exclusion** | | | |
|  | **ICD-8 (1969-1986)** | **ICD-9 (1987-1996)** | **ICD-10 (1997-)** |
| **Other Intellectual disability** | 310, 312-315 | 318, 319 | F71-F73, F78,F79 |
| **Chromosomal abnormalities** | 312.5, 315.5, 759.3-759.5, 759.83 | 758 | Q90-Q99 |

**Table S3.** Age of first event of psychiatric disorder and substance use-related problems in individuals with mild intellectual disability, matched reference individuals from the general population, and full siblings.

| **Psychiatric disorder/**  **Substance use-related problem** | **Individuals with MID** | | | **Reference Individuals** | | | **Full siblings** | | |
| --- | --- | --- | --- | --- | --- | --- | --- | --- | --- |
|  | n | Median | IQR | n | Median | IQR | n | Median | IQR |
| **Anxiety disorder** | 3375 | 20.24 | 7.67 [16.75, 24.42] | 58020 | 20.82 | 7.08 [17.58, 24.66] | 1944 | 21.15 | 8.08 [17.61, 25.69] |
| **Major depressive disorder** | 1966 | 20.93 | 7.62 [17.71, 25.33] | 39194 | 20.88 | 7.06 [17.74, 24.80] | 1218 | 21.58 | 8.11 [17.83, 25.94] |
| **Bipolar disorder** | 383 | 22.00 | 9.10 [18.32, 27.42] | 5479 | 23.00 | 7.11 [19.73, 26.84] | 174 | 23.87 | 8.57 [19.92, 28.49] |
| **Psychotic disorder** | 794 | 21.69 | 7.36 [18.28, 25.63] | 3969 | 21.99 | 6.21 [19.27, 25.49] | 162 | 22.08 | 6.86 [18.90, 25.75] |
| **ADHD** | 4972 | 13.00 | 7.96 [9.49, 17.44] | 32279 | 15.48 | 8.62 [11.69, 20.31] | 1276 | 15.08 | 8.46 [11.23, 19.69] |
| **ASD** | 3437 | 13.73 | 9.67 [8.72, 18.39] | 12065 | 15.92 | 7.99 [12.10, 20.09] | 505 | 16.08 | 8.31 [12.20, 20.51] |
| **Alcohol use disorder** | 1077 | 20.94 | 6.71 [18.11, 24.82] | 26778 | 19.03 | 5.76 [16.50, 22.26] | 824 | 19.46 | 6.19 [16.96, 23.14] |
| **Drug use disorder** | 732 | 22.14 | 6.48 [19.32, 25.80] | 15027 | 20.95 | 5.36 [18.51, 23.87] | 472 | 21.29 | 6.17 [18.47, 24.64] |
| **Alcohol-related somatic disorder** | 15 | 27.41 | 7.24 [22.39, 29.63] | 198 | 22.70 | 6.36 [19.78, 26.14] | 4 | 20.58 | 3.37 [19.26, 22.63] |
| **Substance-use related crime** | 933 | 20.60 | 4.67 [18.52, 23.18] | 34567 | 20.21 | 4.41 [18.19, 22.60] | 981 | 20.52 | 4.95 [18.50, 23.45] |
| **Substance-use related death** | 20 | 28.55 | 6.45 [25.75, 32.21] | 692 | 23.98 | 6.40 [21.06, 27.47] | 23 | 23.98 | 7.84 [22.37, 30.21] |

**Table S4.** Total number of full siblings in each family cluster including index individual.

|  | 1 | 2 | 3 | 4 | 5 | 6+ |
| --- | --- | --- | --- | --- | --- | --- |
| n (%) | 6302 (34.4) | 7250 (39.6) | 3283 (17.9) | 1017 (5.6) | 277 (1.5) | 178 (1.0) |

**Table S5** Incidence rates and risk estimates by substance use-related problem (SUP), and by total and sex, in individuals with mild intellectual disability (MID) compared to matched reference individuals from the general population, and compared to full siblings.

| **Substance use-**  **related problem** | **Population comparison** | | | | **Sibling comparison** | | | |
| --- | --- | --- | --- | --- | --- | --- | --- | --- |
|  | **MID Individuals, n (incidence rate^1^)** | **Reference individuals, n (incidence rate^1^)** | **Crude Model, HR (95% CI) ^2^** | **Adjusted Model, HR (95% CI) ^3^** | **MID Individuals, n (incidence rate^1^)** | **Full siblings, n (incidence rate^1^)** | **Crude Model, HR (95% CI) ^4^** | **Adjusted Model, HR (95% CI) ^5^** |
| **Alcohol use disorder** | 1,077 (2.5) | 26,778 (1.2) | 2.04 [1.92, 2.17] | 1.84 [1.73, 1.96] | 666 (2.4) | 824 (1.8) | 1.35 [1.20, 1.52] | 1.31 [1.16, 1.47] |
| **Drug use disorder** | 732 (1.7) | 15,027 (0.7) | 2.48 [2.30, 2.67] | 2.11 [1.96, 2.28] | 442 (1.6) | 472 (1.0) | 1.64 [1.41, 1.92] | 1.59 [1.37, 1.86] |
| **Alcohol-related somatic disorder** | 15 (0.0) | 198 (0.0) | 3.83 [2.26, 6.47] | 3.43 [2.01, 5.84] | 11 (0.0) | 4 (0.0) | - | - |
| **Substance-use related crime** | 933 (2.2) | 34,567 (1.6) | 1.37 [1.28, 1.46] | 1.13 [1.06, 1.21] | 583 (2.1) | 981 (2.1) | 0.98 [0.87, 1.11] | 0.88 [0.77, 1.01] |
| **Substance-use related death** | 20 (0.0) | 692 (0.0) | 1.45 [0.93, 2.25] | 1.20 [0.77, 1.87] | 9 (0.0) | 23 (0.0) | 0.67 [0.28, 1.58] | 1.08 [0.38, 3.09] |
| **Males** | | | | | | | | |
| **Alcohol use disorder** | 650 (2.6) | 15,700 (1.3) | 2.11 [1.95, 2.28] | 1.90 [1.75, 2.05] | 408 (2.5) | 479 (1.8) | 1.35 [1.16, 1.57] | 1.27 [1.05, 1.55] |
| **Drug use disorder** | 434 (1.8) | 9,965 (0.8) | 2.21 [2.01, 2.44] | 1.88 [1.70, 2.07] | 261 (1.6) | 268 (1.0) | 1.61 [1.32, 1.97] | 1.38 [1.07, 1.78] |
| **Alcohol-related somatic disorder** | 10 (0.0) | 154 (0.0) | 3.25 [1.71, 6.16] | 2.98 [1.56, 5.68] | 7 (0.0) | 0 (0.0) | - | - |
| **Substance-use related crime** | 785 (3.2) | 29,717 (2.4) | 1.34 [1.25, 1.44] | 1.11 [1.03, 1.19] | 489 (3.0) | 571 (2.1) | 1.47 [1.27, 1.69] | 0.85 [0.72, 1.01] |
| **Substance-use related death** | 14 (0.1) | 569 (0.0) | 1.23 [0.72, 2.09] | 1.02 [0.60, 1.73] | 6 (0.0) | 11 (0.0) | 0.93 [0.32, 2.72] | 0.63 [0.15, 2.62] |
| **Females** | | | | | | | | |
| **Alcohol use disorder** | 427 (2.3) | 11,078 (1.2) | 1.95 [1.77, 2.15] | 1.76 [1.60, 1.94] | 258 (2.2) | 345 (1.8) | 1.34 [1.12, 1.62] | 1.46 [1.15, 1.86] |
| **Drug use disorder** | 298 (1.6) | 5,062 (0.6) | 3.00 [2.67, 3.37] | 2.59 [2.31, 2.92] | 181 (1.5) | 204 (1.1) | 1.69 [1.34, 2.14] | 2.17 [1.56, 3.01] |
| **Alcohol-related somatic disorder** | 5 (0.0) | 44 (0.0) | - | - | 4 (0.0) | 4 (0.0) | - | - |
| **Substance-use related crime** | 148 (0.8) | 4,850 (0.5) | 1.53 [1.30, 1.81] | 1.29 [1.09, 1.52] | 94 (0.8) | 410 (2.1) | 0.36 [0.28, 0.46] | 1.18 [0.81, 1.71] |
| **Substance-use related death** | 6 (0.0) | 123 (0.0) | 2.43 [1.07, 5.52] | 2.07 [0.90, 4.74] | 3 (0.0) | 12 (0.1) | - | - |

^1^ Incidence rate per 1000 person-years.

^2^ Cox regression model with matched group as the stratum.

^3^ Additionally adjusted for parental educational attainment.

^4^ Cox regression model with family cluster as the stratum.

^5^ Additionally adjusted for sex and birth-cohort.

**Table S6.** Incidence rates and risk estimates of substance use-related problem (SUP) by psychiatric disorder, by specific SUP, in individuals with mild intellectual disability (MID) compared to matched reference individuals from the general population, and compared to full siblings.

| **Substance use-**  **related problem** | **Population comparison** | | | | **Sibling comparison** | | | |
| --- | --- | --- | --- | --- | --- | --- | --- | --- |
|  | **MID Individuals, n (incidence rate^1^)** | **Reference individuals, n (incidence rate^1^)** | **Crude Model, HR (95% CI) ^2^** | **Adjusted Model, HR (95% CI) ^3^** | **MID Individuals, n (incidence rate^1^)** | **Full siblings, n (incidence rate^1^)** | **Crude Model, HR (95% CI) ^4^** | **Adjusted Model, HR (95% CI) ^5^** |
| **Anxiety disorder** | | | | | | | | |
| **Alcohol use disorder** | 582 (6.7) | 5,505 (1.2) | 5.66 [5.19, 6.17] | 5.09 [4.66, 5.56] | 346 (6.3) | 232 (2.5) | 2.58 [2.13, 3.12] | 2.57 [2.11, 3.12] |
| **Drug use disorder** | 482 (5.5) | 3,056 (0.7) | 8.47 [7.69, 9.33] | 7.17 [6.49, 7.92] | 302 (5.4) | 149 (1.6) | 3.52 [2.79, 4.44] | 3.52 [2.77, 4.47] |
| **Alcohol-related somatic disorder** | 9 (0.1) | 36 (0.0) | 12.77 [6.14, 26.56] | 14.53 [6.56, 32.18] | 5 (0.1) | 3 (0.0) | - | - |
| **Substance-use related crime** | 367 (4.2) | 6,518 (1.4) | 2.98 [2.68, 3.31] | 2.46 [2.21, 2.74] | 240 (4.3) | 280 (3.0) | 1.43 [1.17, 1.75] | 1.63 [1.29, 2.04] |
| **Substance-use related death** | 13 (0.1) | 149 (0.0) | 4.34 [2.46, 7.64] | 3.45 [1.93, 6.16] | 6 (0.1) | 11 (0.1) | 0.80 [0.26, 2.45] | 0.87 [0.24, 3.22] |
| **Major depressive disorder** | | | | | | | | |
| **Alcohol use disorder** | 398 (7.7) | 3,280 (1.2) | 6.59 [5.93, 7.31] | 5.90 [5.31, 6.56] | 238 (7.3) | 154 (2.8) | 2.86 [2.25, 3.62] | 2.90 [2.27, 3.71] |
| **Drug use disorder** | 335 (6.4) | 1,823 (0.7) | 9.93 [8.83, 11.17] | 8.41 [7.46, 9.48] | 216 (6.5) | 96 (1.7) | 4.48 [3.33, 6.03] | 4.42 [3.27, 5.98] |
| **Alcohol-related somatic disorder** | 6 (0.1) | 22 (0.0) | 14.20 [5.73, 35.18] | 19.65 [7.28, 53.02] | 3 (0.1) | 1 (0.0) | - | - |
| **Substance-use related crime** | 228 (4.3) | 3,829 (1.4) | 3.16 [2.77, 3.62] | 2.66 [2.33, 3.05] | 144 (4.3) | 163 (3.0) | 1.39 [1.08, 1.80] | 1.53 [1.14, 2.05] |
| **Substance-use related death** | 3 (0.1) | 78 (0.0) | 1.91 [0.60, 6.06] | 1.55 [0.49, 4.95] | 2 (0.1) | 4 (0.1) | - | - |
| **Bipolar disorder** | | | | | | | | |
| **Alcohol use disorder** | 77 (7.5) | 628 (1.2) | 6.62 [5.22, 8.40] | 6.14 [4.83, 7.81] | 53 (7.9) | 33 (2.9) | 2.73 [1.65, 4.51] | 2.71 [1.61, 4.56] |
| **Drug use disorder** | 94 (9.1) | 340 (0.6) | 15.62 [12.38, 19.70] | 14.08 [11.10, 17.87] | 60 (8.9) | 20 (1.7) | 5.43 [2.98, 9.90] | 6.11 [3.20, 11.68] |
| **Alcohol-related somatic disorder** | 2 (0.2) | 6 (0.0) | - | - | 1 (0.1) | 0 (0.0) | - | - |
| **Substance-use related crime** | 45 (4.3) | 771 (1.4) | 3.06 [2.26, 4.13] | 2.76 [2.03, 3.73] | 32 (4.7) | 30 (2.6) | 2.04 [1.12, 3.71] | 2.03 [1.09, 3.78] |
| **Substance-use related death** | 0 (0.0) | 22 (0.0) | - | - | 0 (0.0) | 0 (0.0) | - | - |
| **Psychotic disorder** | | | | | | | | |
| **Alcohol use disorder** | 139 (6.1) | 1,284 (1.1) | 5.87 [4.93, 7.00] | 5.35 [4.48, 6.40] | 82 (5.8) | 63 (2.6) | 2.54 [1.74, 3.72] | 2.51 [1.70, 3.70] |
| **Drug use disorder** | 179 (7.8) | 788 (0.7) | 12.68 [10.76, 14.94] | 10.94 [9.25, 12.93] | 107 (7.6) | 49 (2.0) | 4.72 [3.11, 7.18] | 5.17 [3.29, 8.14] |
| **Alcohol-related somatic disorder** | 4 (0.2) | 13 (0.0) | - | - | 3 (0.2) | 1 (0.0) | - | - |
| **Substance-use related crime** | 107 (4.6) | 1,936 (1.6) | 2.97 [2.45, 3.62] | 2.56 [2.10, 3.11] | 67 (4.7) | 77 (3.2) | 1.39 [0.95, 2.04] | 1.40 [0.91, 2.14] |
| **Substance-use related death** | 7 (0.3) | 41 (0.0) | 8.65 [3.88, 19.32] | 8.44 [3.67, 19.44] | 2 (0.1) | 2 (0.1) | - | - |
| **ADHD** | | | | | | | | |
| **Alcohol use disorder** | 460 (4.4) | 6,093 (1.2) | 3.98 [3.62, 4.37] | 3.60 [3.27, 3.96] | 271 (4.2) | 234 (2.3) | 2.09 [1.69, 2.57] | 2.05 [1.65, 2.55] |
| **Drug use disorder** | 400 (3.8) | 3,478 (0.7) | 6.05 [5.45, 6.71] | 5.17 [4.66, 5.75] | 245 (3.8) | 131 (1.3) | 3.60 [2.76, 4.70] | 3.60 [2.74, 4.72] |
| **Alcohol-related somatic disorder** | 6 (0.1) | 41 (0.0) | 7.47 [3.17, 17.62] | 6.33 [2.61, 15.32] | 3 (0.0) | 1 (0.0) | - | - |
| **Substance-use related crime** | 448 (4.3) | 8,401 (1.6) | 2.81 [2.56, 3.09] | 2.35 [2.13, 2.59] | 274 (4.2) | 254 (2.5) | 1.84 [1.50, 2.26] | 1.54 [1.23, 1.92] |
| **Substance-use related death** | 8 (0.1) | 146 (0.0) | 2.72 [1.34, 5.55] | 2.24 [1.09, 4.61] | 1 (0.0) | 4 (0.0) | - | - |
| **ASD** | | | | | | | | |
| **Alcohol use disorder** | 176 (2.4) | 4,027 (1.1) | 2.23 [1.92, 2.59] | 2.10 [1.81, 2.45] | 113 (2.4) | 124 (1.7) | 1.67 [1.23, 2.27] | 1.66 [1.21, 2.26] |
| **Drug use disorder** | 149 (2.1) | 2,330 (0.6) | 3.26 [2.76, 3.84] | 2.96 [2.51, 3.50] | 93 (2.0) | 81 (1.1) | 2.17 [1.51, 3.12] | 2.12 [1.46, 3.09] |
| **Alcohol-related somatic disorder** | 4 (0.1) | 32 (0.0) | - | - | 2 (0.0) | 0 (0.0) | - | - |
| **Substance-use related crime** | 109 (1.5) | 5,415 (1.5) | 1.00 [0.83, 1.21] | 0.90 [0.75, 1.09] | 69 (1.5) | 137 (1.9) | 0.76 [0.53, 1.07] | 0.59 [0.40, 0.87] |
| **Substance-use related death** | 2 (0.0) | 79 (0.0) | - | - | 1 (0.0) | 5 (0.1) | - | - |

^1^ Incidence rate per 1000 person-years.

^2^ Cox regression model with matched group as the stratum.

^3^ Additionally adjusted for parental educational attainment.

^4^ Cox regression model with family cluster as the stratum.

^5^ Additionally adjusted for sex and birth-cohort.

**REFERENCES**

Arvidsson, J. (2016). *Sysselsättning och social rättvisa : En nationell registerstudie om 12 269 unga vuxna med intellektuell funktionsnedsättning.* (Doctoral thesis, comprehensive summary). Halmstad University Press, Halmstad. Retrieved from <http://urn.kb.se/resolve?urn=urn:nbn:se:hh:diva-30768> DiVA database. (19)

Brooke, H. L., Talbäck, M., Hörnblad, J., Johansson, L. A., Ludvigsson, J. F., Druid, H., . . . Ljung, R. (2017). The Swedish cause of death register. *European journal of epidemiology, 32*(9), 765-773. doi:10.1007/s10654-017-0316-1

Cnattingius, S., Ericson, A., Gunnarskog, J., & Källén, B. (1990). A Quality Study of a Medical Birth Registry. *Scandinavian Journal of Social Medicine, 18*(2), 143-148. doi:10.1177/140349489001800209

Idring, S., Rai, D., Dal, H., Dalman, C., Sturm, H., Zander, E., . . . Magnusson, C. (2012). Autism spectrum disorders in the Stockholm Youth Cohort: design, prevalence and validity. *PloS one, 7*(7), e41280. Retrieved from <https://www.ncbi.nlm.nih.gov/pmc/articles/PMC3401114/pdf/pone.0041280.pdf>

Ludvigsson, J. F., Almqvist, C., Bonamy, A. K., Ljung, R., Michaelsson, K., Neovius, M., . . . Ye, W. (2016). Registers of the Swedish total population and their use in medical research. *European journal of epidemiology, 31*(2), 125-136. doi:10.1007/s10654-016-0117-y

Ludvigsson, J. F., Andersson, E., Ekbom, A., Feychting, M., Kim, J.-L., Reuterwall, C., . . . Olausson, P. O. (2011). External review and validation of the Swedish national inpatient register. *BMC public health, 11*(1), 450.

Ludvigsson, J. F., Svedberg, P., Olén, O., Bruze, G., & Neovius, M. (2019). The longitudinal integrated database for health insurance and labour market studies (LISA) and its use in medical research. *European journal of epidemiology, 34*(4), 423-437. Retrieved from <https://www.ncbi.nlm.nih.gov/pmc/articles/PMC6451717/pdf/10654_2019_Article_511.pdf>

Långström, N., Grann, M., Ruchkin, V., Sjöstedt, G., & Fazel, S. (2008). Risk Factors for Violent Offending in Autism Spectrum Disorder: A National Study of Hospitalized Individuals. *Journal of Interpersonal Violence, 24*(8), 1358-1370. doi:10.1177/0886260508322195

Wettermark, B., Hammar, N., MichaelFored, C., Leimanis, A., Otterblad Olausson, P., Bergman, U., . . . Rosén, M. (2007). The new Swedish Prescribed Drug Register—Opportunities for pharmacoepidemiological research and experience from the first six months. *Pharmacoepidemiology and Drug Safety, 16*(7), 726-735. doi:<https://doi.org/10.1002/pds.1294>
